# Supplementary material for: Magnesium ions mediate ligand binding and conformational transition of the SAM/SAH riboswitch
Source: Commun Biol. 2023 Jul 31;6:791. doi: 10.1038/s42003-023-05175-5 (PMC10390503; doi:10.1038/s42003-023-05175-5)
Supplement: Supplementary file 2 — Supplementary Materials [file 42003_2023_5175_MOESM2_ESM.pdf]

Magnesium ions mediate ligand binding and conformational transition of the SAM/SAH riboswitch

Guodong Hu[1, 2] and Huan-Xiang Zhou[2, 3,*]

[1]Shandong Key Laboratory of Biophysics, Dezhou University, Dezhou 253023, China

[2]Department of Chemistry, University of Illinois Chicago, Chicago, IL 60607, USA

[3]Department of Physics, University of Illinois Chicago, Chicago, IL 60607, USA

*E-mail: hzhou43@uic.edu

Supplementary Materials

Supplementary Table 1. Systems and molecular dynamics simulation lengths.

Systems	# of Mg^{2+} ions	Models	# of rep × MD length (μs)	Sum (μs)
SAH / SAM	41 (Leap)	10	4×1 = 4	80
Apo	41 (Leap)	1	4×1 = 4	4
SAH / SAM/ Apo	25 (MCTBI)	1	4×1 = 4	12
SAH / SAM / Apo	21 (Leap)	1	4×1 = 4	12
SAH / SAM/ Apo	0	1	4×1 = 4	12

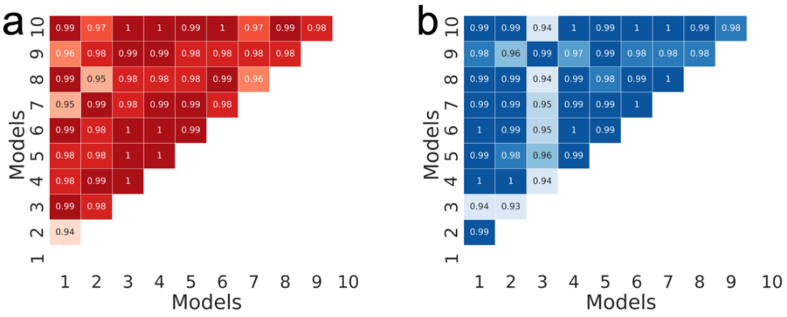

Supplementary Figure 1. Correlations of nucleotide-ligand interaction energies between starting NMR models. (a) SAH. (b) SAM.

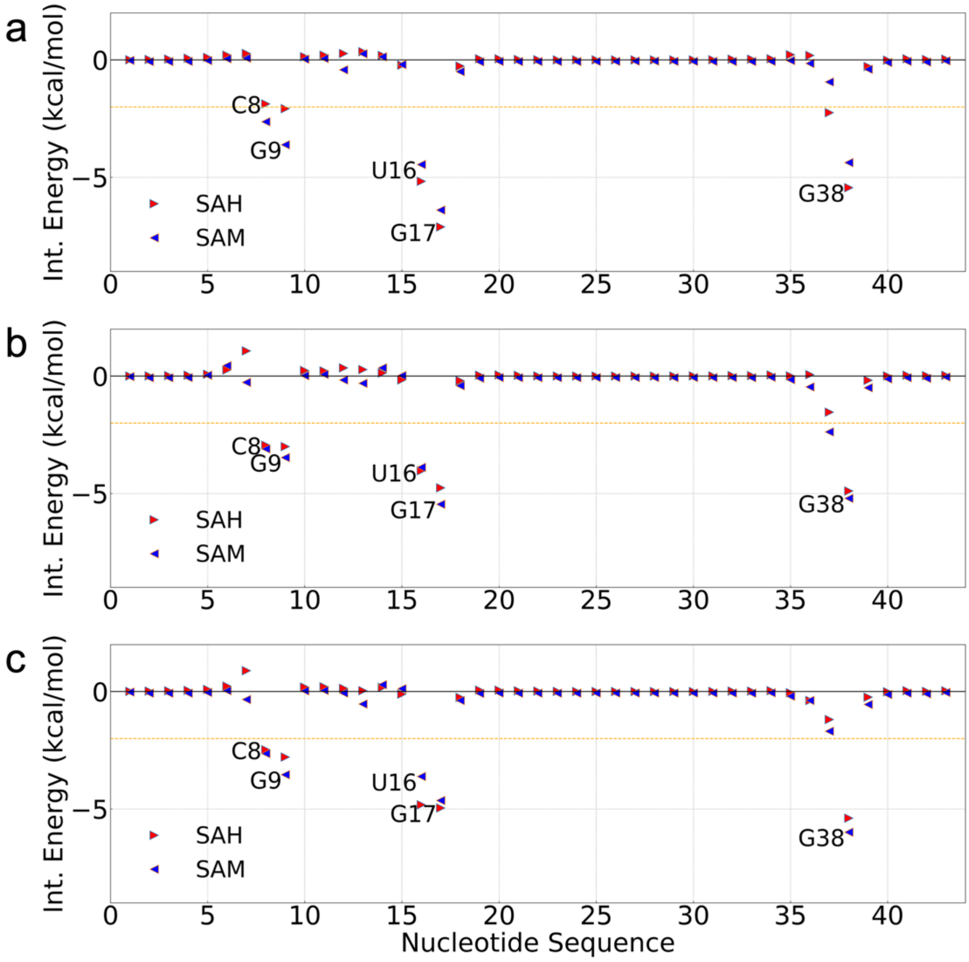

Supplementary Figure 2. Interaction energies of individual nucleotides with the ligands. (a) Simulations started with the MCTBI protocol. (b) Simulations when free of Mg^{2+}. (c) Simulations started with the Leap(21) protocol. A horizontal line is drawn at interaction energy = -2.0 kcal/mol.

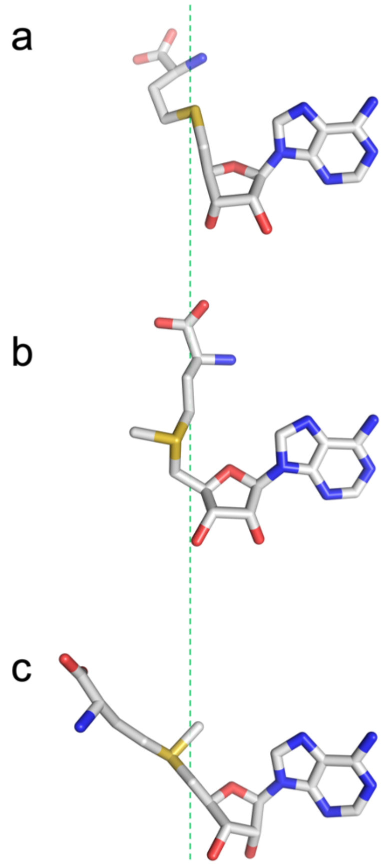

Supplementary Figure 3. Conformations sampled by ligands in the bound state. (a) U-shape conformation of SAH. (b) U-shape conformation of SAM. (c) L-shape conformation of SAM. The vertical line roughly demarcates the groove entrance; the U16 and G17 bases are to the right of the vertical line.

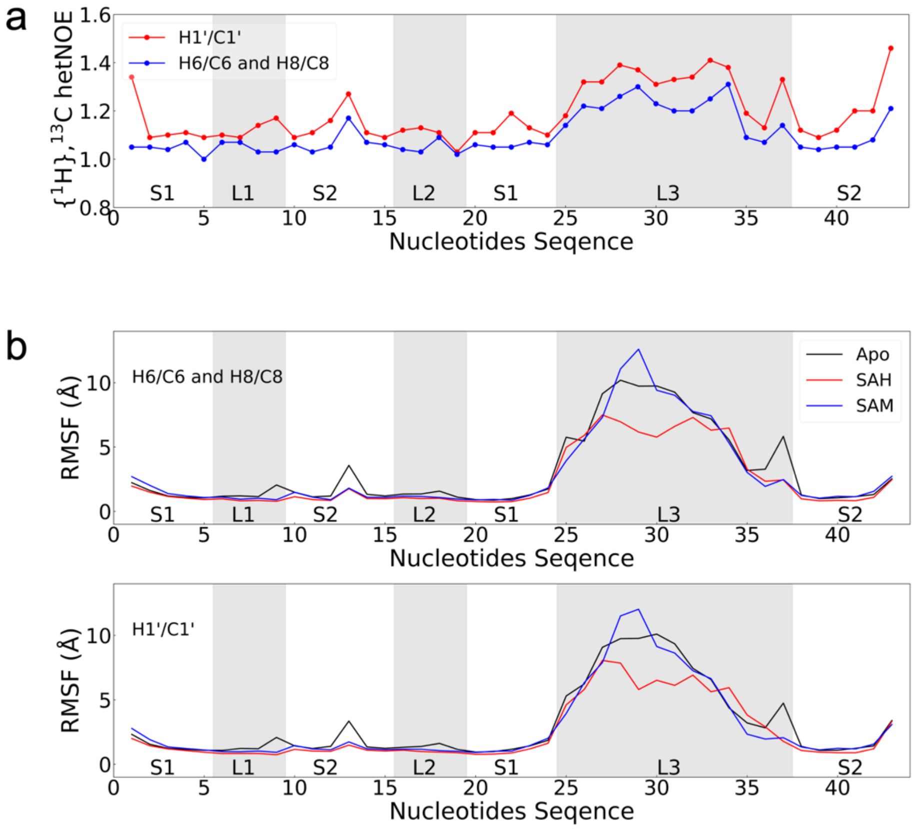

Supplementary Figure 4. Flexibility profiles determined by NMR and MD simulations. (a) ^{1}H-^{13}C heteronuclear Overhauser effects in the SAH-bound form, replotted using data reported by Weickhmann *et al.* [*Nucleic Acids Res* **47**, 2654-2665 (2019)]. (b) RMSFs from MD simulations with the MCTBI protocol.

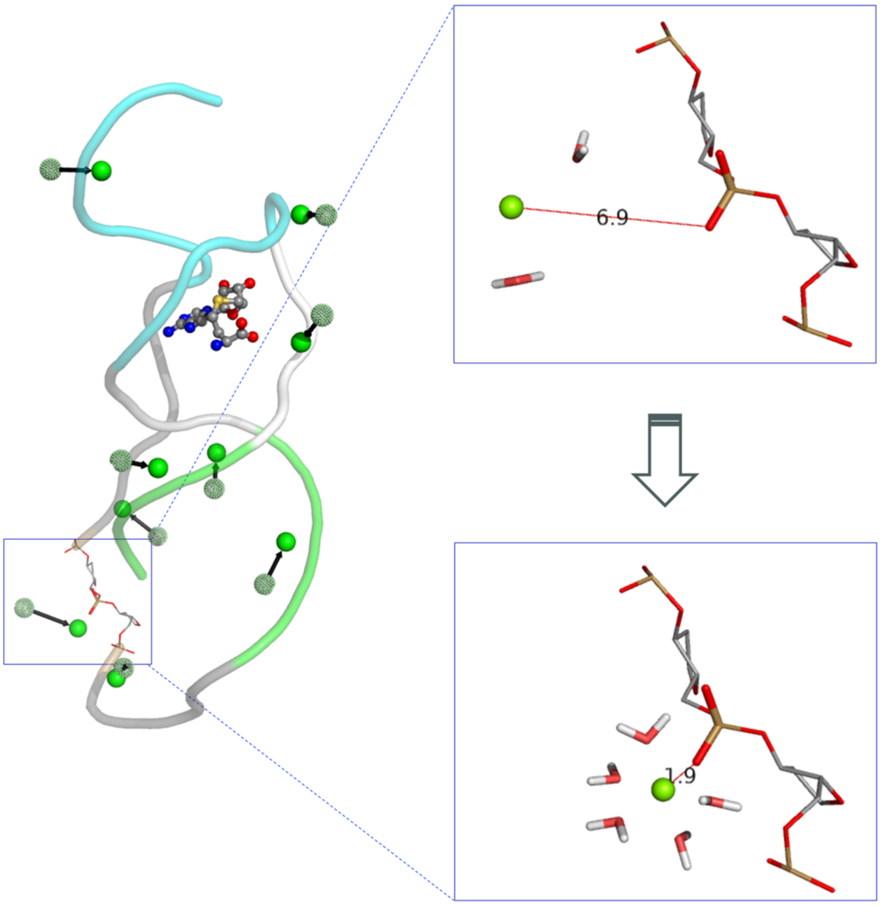

Supplementary Figure 5. Movement of a Mg^{2+} ion into an inner-shell position. The left image shows a snapshot of the SAH-bound RNA in the simulations started with the Leap(21) protocol. Spheres with dot and smooth surfaces represent initial and final positions of inner-shell Mg^{2+} ions. The boxed region highlights the Mg^{2+} ion that started from the largest initial distance from a phosphate but moved to an inner-shell position. This region is enlarged on the right, with the top image showing the initial structure and the bottom image showing the structure at 2 ps into the heating stage of the simulations. Water molecules with any atom within 2.5 Å of the Mg^{2+} ion are shown as stick. A movie showing the movement of this Mg^{2+} ion is shown in Supplementary Movie 1.

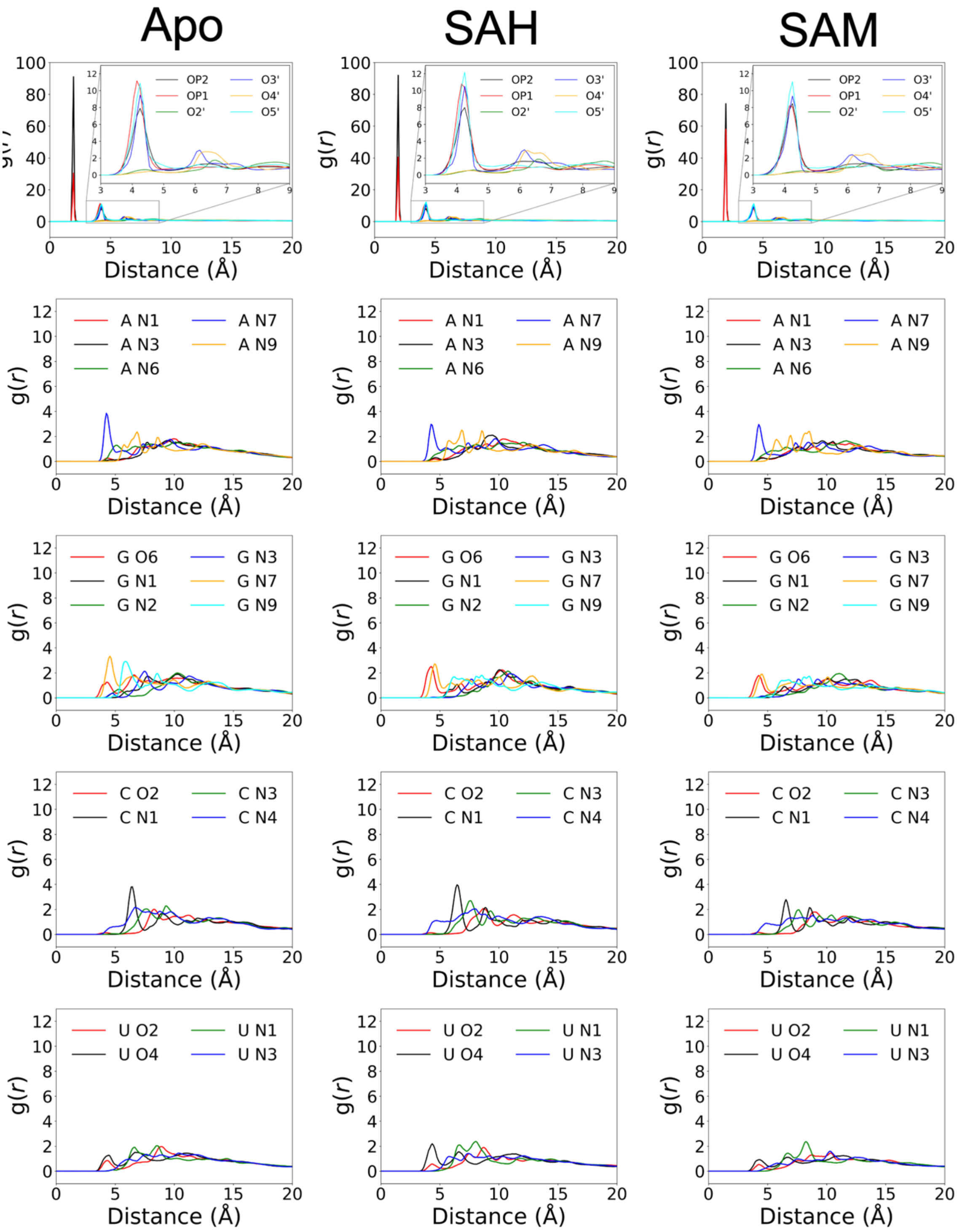

Supplementary Figure 6. Radial distribution functions of Mg^{2+} ions around backbone and base atoms in the apo and SAH and SAM-bound forms.

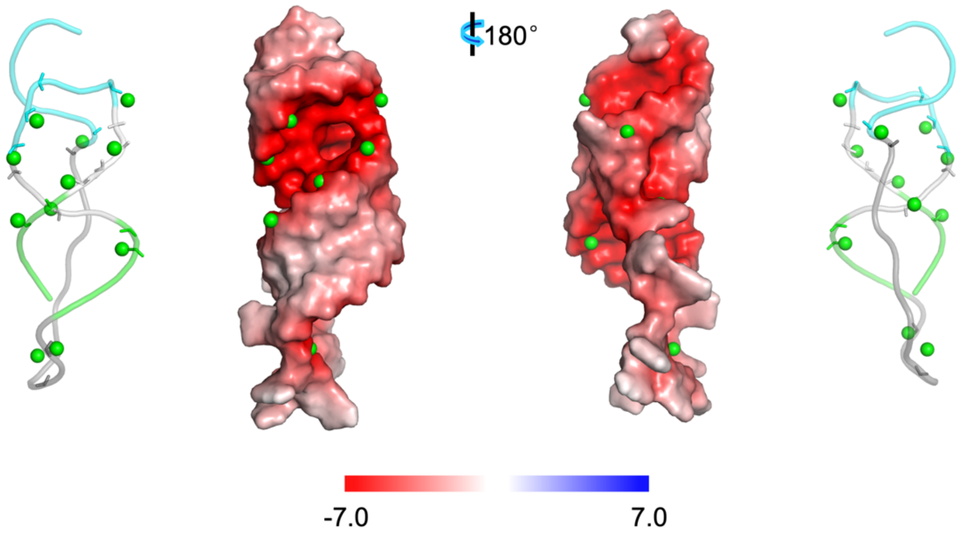

Supplementary Figure 7. Electrostatic potential surface of the RNA, with a conformation taken from the MD simulations of the SAM-bound form. The left two images show the ”front” view and the right two images show the “back” view. The electrostatic potential was calculated using APBS [N. A. Baker, D. Sept, S. Joseph, M. J. Holst and J. A. McCammon, *Proc Natl Acad Sci U S A* 2001 **98**, 10037-10041 (2001)] on the RNA molecule only. The inner-shell Mg^{2+} ions are added back to the cartoon representation and to the electrostatic potential surface.

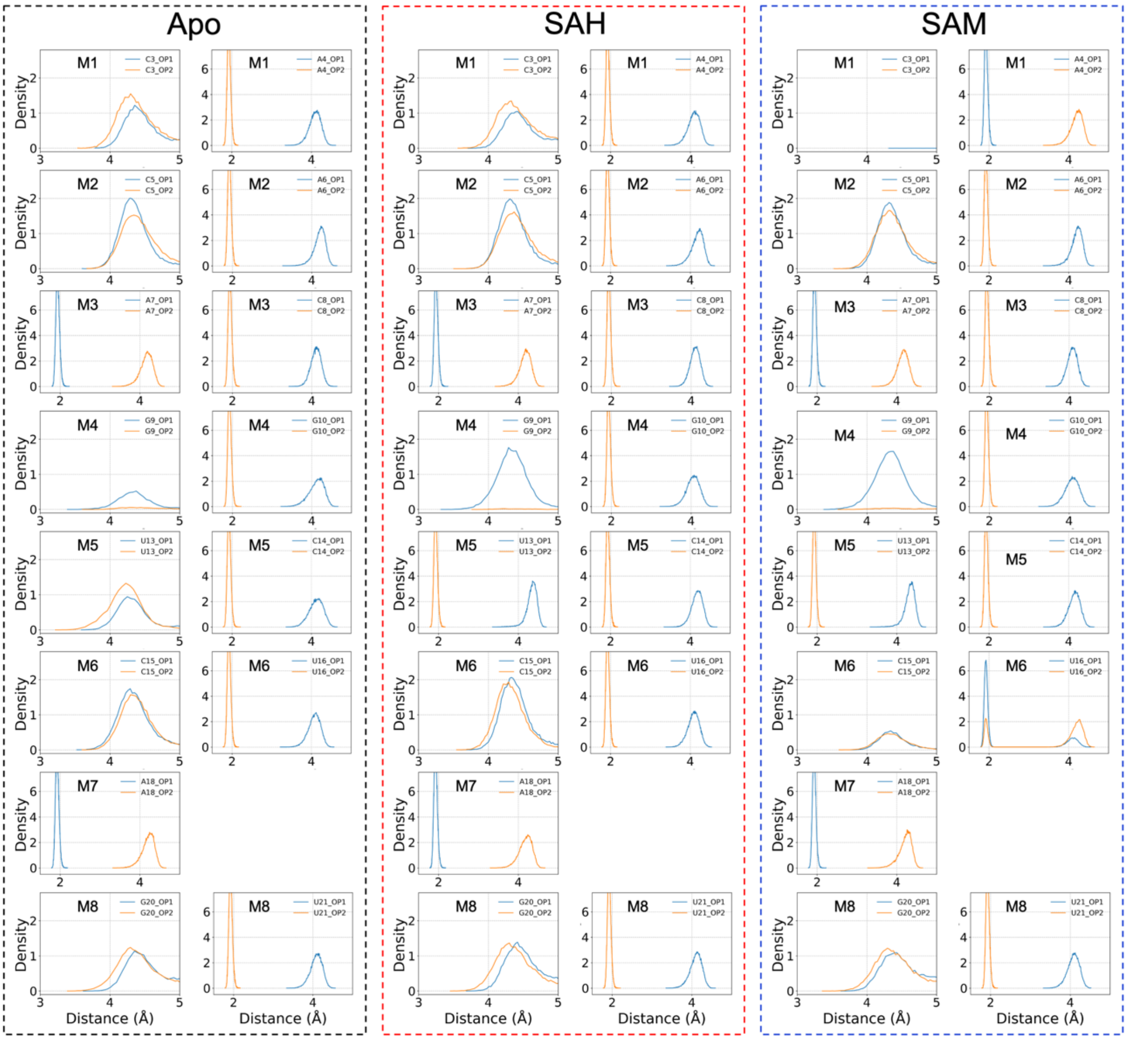

Supplementary Figure 8. The distributions of distances between phosphate OP1 and OP2 atoms and conserved Mg^{2+} ions.

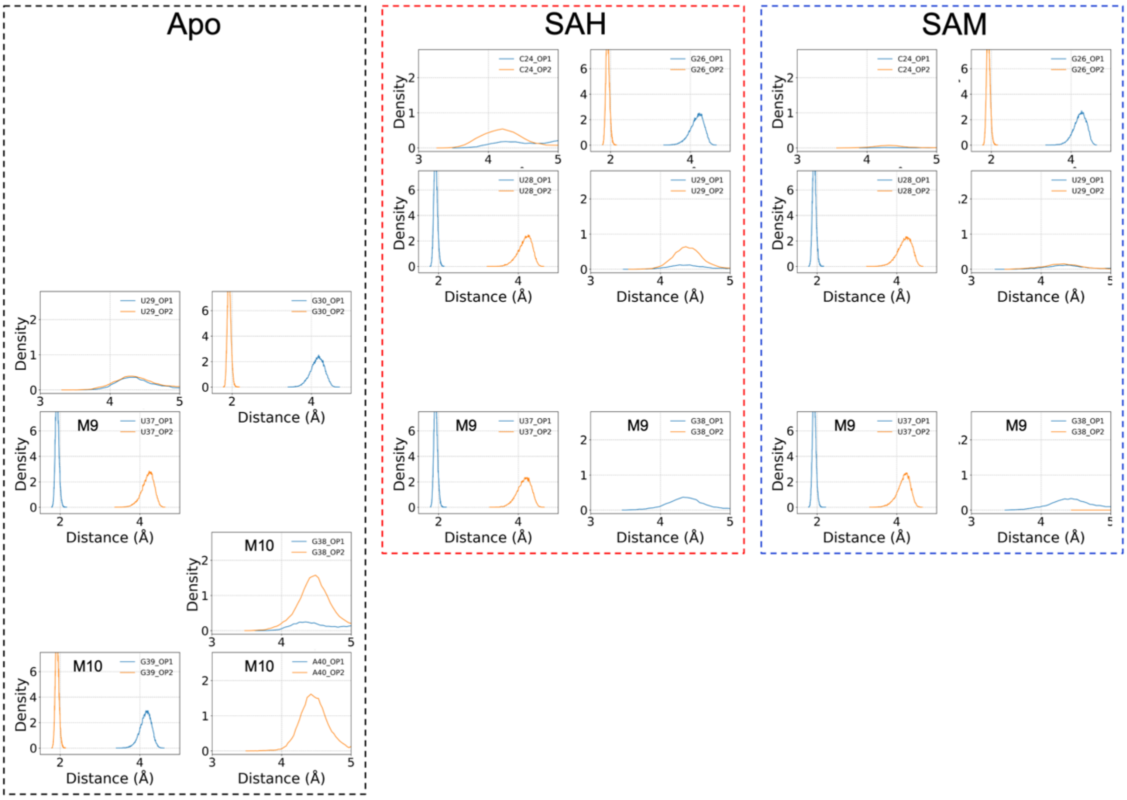

Supplementary Figure 9. The distributions of distances between phosphate OP1 and OP2 atoms and Mg^{2+} ions showing distinction between the apo and liganded forms.

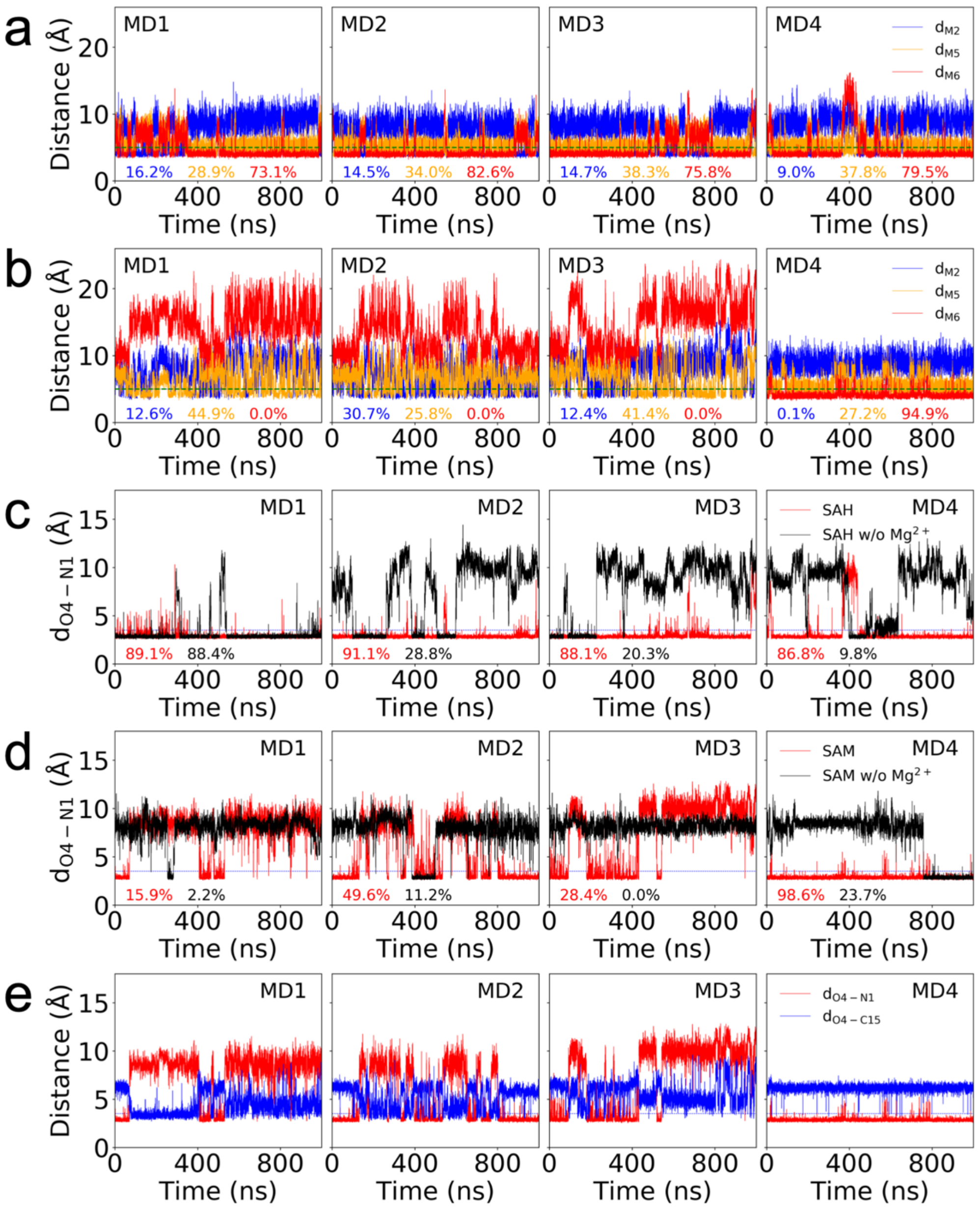

Supplementary Figure 10. Traces of interatomic distances in four replicate simulations. (a) Distances of the SAH carboxy moiety to three inner-shell Mg^{2+} ions. A horizontal line is drawn at 5 Å to indicate the cutoff for outer-shell coordination. The fractions of frames with outer-shell coordination are shown as percentages. (b) Corresponding results for the SAM-bound form. (c) The O4-N1 distances in the SAH-bound form without (labeled as "w/o") or with saturating Mg^{2+}. A horizontal line at 3.5 Å indicates the cutoff for hydrogen bond formation. The fractions of frames forming the hydrogen bond are shown as percentages. (d) Corresponding results for the SAM-bound form. (e) The O4-N1 and O4-C15 distances for the SAM-bound form with saturating Mg^{2+}.

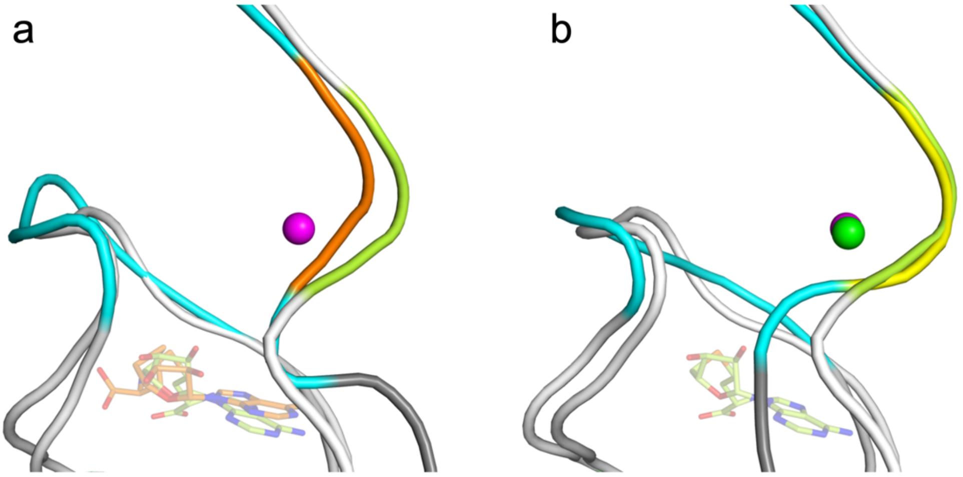

Supplementary Figure 11. Comparison of the G39-A40-G41 backbone shapes between 6HAG, 6LY5, and a representative structure from the simulations of the apo form. (a) Superposition of 6HAG and 6LY5. G39-A40-G41 is shown in orange for 6HAG and limon for 6LY5; the ligands are shown with carbon atoms in the same two colors; note the different orientations of the carboxy moiety. A Na^+ ion from 6HAG is shown as a magenta sphere. (b) Similar comparison but 6HAG is replaced by the MD structure for the apo form and the color changed from orange to yellow. M10 is shown as a green sphere.

Supplementary Movie 1. Movement of a Mg^{2+} ion into an inner-shell position, during the energy minimization and first 2 ps into the heating stage of the simulations. The initial and final frames of this movie and the caption are found in Supplementary Figure 5.
