# Supplementary material for: Magnesium ions mediate ligand binding and conformational transition of the SAM/SAH riboswitch
Source: Commun Biol. 2023 Jul 31;6:791. doi: 10.1038/s42003-023-05175-5 (PMC10390503; doi:10.1038/s42003-023-05175-5)
Supplement: Supplementary file 3 — Description of Additional Supplementary Files [file 42003_2023_5175_MOESM3_ESM.pdf]

Description of Additional Supplementary Files

File name: Supplementary Movie 1

Description: Movement of a Mg^{2+} ion into an inner-shell position, during the energy minimization and first 2 ps into the heating stage of the simulations. The initial and final frames of this movie and the caption are found in Supplementary Figure 5.
